# Respiratory depression in the post-anesthesia care unit: Mayo Clinic experience

**DOI:** 10.17305/bjbms.2020.4816

**Published:** 2021-04

**Authors:** Mariana L. Laporta, Juraj Sprung, Toby N. Weingarten

**Affiliations:** Department of Anesthesiology and Perioperative Medicine, Mayo Clinic, Rochester, Minnesota, USA

**Keywords:** Respiratory depression, post-anesthesia care unit, ERAS

## Abstract

The anesthesia recovery is a complex physiologic process as systems recover from the effects of surgery and anesthesia. Inadequate recovery of respiratory physiology can lead to severe hypoxemia-induced end-organ damage and even death. Emerging evidence suggests that signs of respiratory depression during early anesthesia recovery may portend increased risk for future severe adverse events. This article briefly reviews the Mayo Clinic research experience and advances in clinical practice. From the implementation of a step-down model of discharge criteria in the post-anesthesia care unit (PACU), consisting of PACU nurses monitoring patients in predetermined periods for signs of respiratory depression, and delaying PACU discharge for patients who exhibit signs of respiratory depression, and early intervention in high-risk patients. Subsequent studies found that even a single episode of respiratory depression in the PACU was strongly associated with subsequent respiratory complications. Further, patient baseline characteristics found to be associated with respiratory depression included obstructive sleep apnea and low body weight, and surgical factors associated with increased risk included the use of preoperative sustained-release opioids, perioperative gabapentinoid use, higher intraoperative opioids, isoflurane as the volatile anesthetic, and longer surgical duration. Based in part of Mayo Clinic research, the FDA issued a warning in 2019 on gabapentinoids use and respiratory complications, increasing the recommended level of respiratory vigilance in patients using this medication. Understanding the complex nature of postoperative respiratory events requires a range of translational and clinical research and constant update of practice.

## INTRODUCTION

A post-anesthesia care unit (PACU) is a vital part of hospital surgical suites and ambulatory care centers. It may be described as *critical care unit* where the patient’s vital signs are closely observed, postoperative pain management is initiated, and all needed treatments are provided in order to recover patient for the appropriate level of discharge, i.e., to home (outpatients), regular ward, or critical care unit. Recovery from anesthesia may be complex and labor-intense and requires a high level of staffing by nurses with specialty training in PACU management. PACU nurses and attending anesthesiologists must closely monitor patients and promptly correct all altered physiologic homeostasis related to procedures and anesthesia. For example, preexisting pulmonary pathology or obstructive sleep apnea (OSA), and the use of respiratory depressants (opioids) and muscle relaxants may complicate the transition from an anesthetized state with controlled ventilation to awake state with spontaneous breathing. Inadequate recovery of respiratory muscles related to residual effects of muscle relaxants, and depressed respiratory drive by opioids, can lead to complications associated with severe hypoxemia, hypercarbia, cardiovascular complications, or death. Emerging evidence suggests that respiratory depression during immediate anesthesia recovery in PACU may portend increased risk for severe adverse events following PACU discharge, and this is the primary aim of our review.

### Overview of PACU management

Several years ago, the Mayo Clinic instituted a change in discharge criteria for surgical patients in the PACU. This change consisted of implementing a practice where PACU nurses monitor for signs of respiratory depression, and patients experiencing an episode of respiratory depression receive additional attention: 1) delayed discharge from PACU to capture the frequency of respiratory depression, 2) patients who had repeated episodes of respiratory depression receive an elevated level of postoperative monitoring (unit with advanced monitoring capabilities), and 3) very high-risk patients receive positive airway pressure (PAP) therapy (continuous or bilevel positive pressure ventilation, CPAP or BiPAP) [1]. Our subsequent study demonstrated that even a single episode of respiratory depression in the PACU is strongly associated with in-hospital respiratory complications [2]. Risk factors associated with respiratory depression were OSA, preoperative use of sustained-release opioids or gabapentin, higher intraoperative opioid use, use of isoflurane, and longer surgical duration. Based in part of Mayo Clinic research [3-6], the FDA in 2019 issued a warning on gabapentinoids usage and respiratory complications, increasing the recommended level of respiratory vigilance in patients using this medication [7].

### Main issues in post-anesthesia recovery

The recovery following anesthesia and surgery is complex, marked by the readjustment of altered organ physiology. Substantial dysfunction of respiratory physiology occurs following even the most straightforward general anesthetic, in part related to the effects of perioperative medications and mechanical ventilation. Accordingly, during the early phase of anesthetic recovery, potentially life-threatening respiratory complications are not uncommon (e.g., hypoxemia, hypoventilation, airway obstruction [8], and residual neuromuscular blockade resulting in respiratory failure [9]).

### Introduction to PACU

During World War II, many medical practices developed dedicated nursing units to provide high-level of care to patients emerging from anesthesia so that these early complications could be promptly recognized and addressed [10]. These dedicated units became known as post-anesthesia care units (PACU or recovery rooms), and their creation made substantial contributions for increasing the safety of anesthesia and, nowadays, became a standard of care in surgical practices [11]. As surgical and anesthesia practices evolved, there were similar improvements in nursing care for patients admitted to the PACU. One of these innovations was the establishment of PACU discharge criteria. In 1971, Aldrete and Kroulik [12] described five criteria which needed to be met before discharge: the return of consciousness, strength, color (absence of cyanosis), and the ability to cough on command, as well as the return of baseline blood pressure; which represented normalization of neurologic, motor, pulmonary, and cardiovascular physiology. In the 1990s, these criteria were further refined to reflect improvements in physiologic patient monitors (e.g., oxyhemoglobin saturation assessed from pulse oximetry supplanted the use of skin pallor spectrum) [13]. These criteria, in addition to the use of a “goal pain score” and adequate control of postoperative nausea and vomiting, are now widely used throughout the world.

### Shortcomings of widely used PACU discharge criteria: Respiratory depression

These discharge criteria assess the return of pulmonary function based on the patient ability to achieve a predefined level of oxyhemoglobin saturation (often 92%, in some cases while receiving supplemental oxygen) and the ability to “maintain an airway”, which is assessed by the patient’s capacity to cough on command. These existing criteria do not accurately assess the respiratory drive. This is a concern for several reasons. First, decrements in oxyhemoglobin saturations occur promptly in response to hypoventilation in subjects breathing room air [14]. However, the application of supplemental oxygen (even low levels) substantially blunts this response; thus, patients receiving supplemental oxygen can hypoventilate and can be severely hypercarbic, but have clinically acceptable oxyhemoglobin saturation by pulse oximetry. For example, a clinical trial by Fu et al. [14] evaluated the effects of deliberate hypoventilation (by decreasing the minute ventilation 50%) on oxyhemoglobin saturation levels. When the inspired oxygen was maintained at 25%–30%, oxyhemoglobin saturation remained at >95% despite a dramatic increase of arterial CO_2_ levels in response to hypoventilation [14]. At the Mayo Clinic, there are almost universal applications of supplemental oxygen for postsurgical patients upon admission to the PACU, and approximately 40% of those discharged from the PACU to postsurgical wards continue receiving supplemental oxygen. This practice is typical of modern anesthesia practices; however, the implication is that it limits the accuracy of pulse oximeter-assessed oxyhemoglobin saturation to detect hypoventilation. At the same time, the use of perioperative opioid analgesics increased dramatically in the early 2000s in response to the widespread recognition that pain was being inadequately treated and pressure from regulatory agencies and patient advocacy groups to better address perioperative pain (the so-called “decade of pain” and incorporation of “pain” as the *“fifth vital sign”* [15]). For example, the Mayo Clinic Scottsdale practice documented a 60% increase in perioperative opioid dose administration for surgical procedures between the years 2000 and 2002 [16]. While recognition that liberal opioid prescription strategies have resulted in widespread opioid use disorders (the “opioid crisis”) and abundantly relaxed perioperative opioid administration, there has been an emphasis on the perioperative use of other opioid-sparring sedating analgesics, primarily gabapentin and pregabalin, which themselves can depress the respiratory rate when used concomitantly with opioid analgesics [3,6,17,18]. Another critical concern is that OSA and other sleep-breathing disorders are often unrecognized and may have a prevalence as high as 20% among adult surgical patients [19]. This prevalence may be increasing as the rates of obesity (a known risk factor for OSA) among the surgical population have increased dramatically over the last several decades [20].

Postoperative hypoventilation and respiratory arrests can result in catastrophic complications. An analysis of 92 closed claims of opioid-induced respiratory depression reported that 22% resulted in anoxic brain injury and 55% in death and that these suits resulted in a median payout of $217,000 [21]. These respiratory arrests can represent “*failure to rescue*” events, which were defined by Silber et al. [22] as hospital deaths after adverse occurrences such as postsurgical complications. This analysis found that: from patients with known respiratory depression time, 73.4% had it within 2 hours interval of nursing checking, and 62% were noted to have some degree of somnolence before the event; reinforcing the importance of effective continuous monitoring [21]. Because *“failure to rescue”* events are tragic for patients and families, unsettling for the healthcare team, and costly to healthcare systems, an approach to reduce their incidence is highly desirable.

### Mayo Clinic PACU practice

**FIGURE 1:**
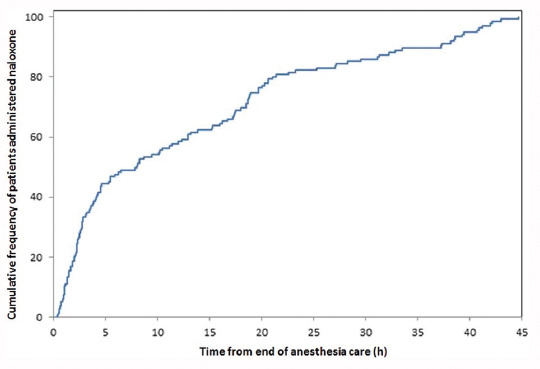
Time from anesthesia end to naloxone administration. With permission from Weingarten et al. [29].

A review of opioid-induced respiratory arrest timing in temporal relationship to PACU discharge demonstrates that a substantial portion happens in the first few hours following PACU discharge (Figure 1) [23]. This temporal relationship suggests that there may be potential signs of early deterioration in the PACU, which could portend these complications. However, as previously discussed, the current widely used PACU discharge criteria do not systematically assess respiratory drive, especially in patients receiving supplemental oxygen, thus hindering an early sign of respiratory physiologic deterioration.

**FIGURE 2:**
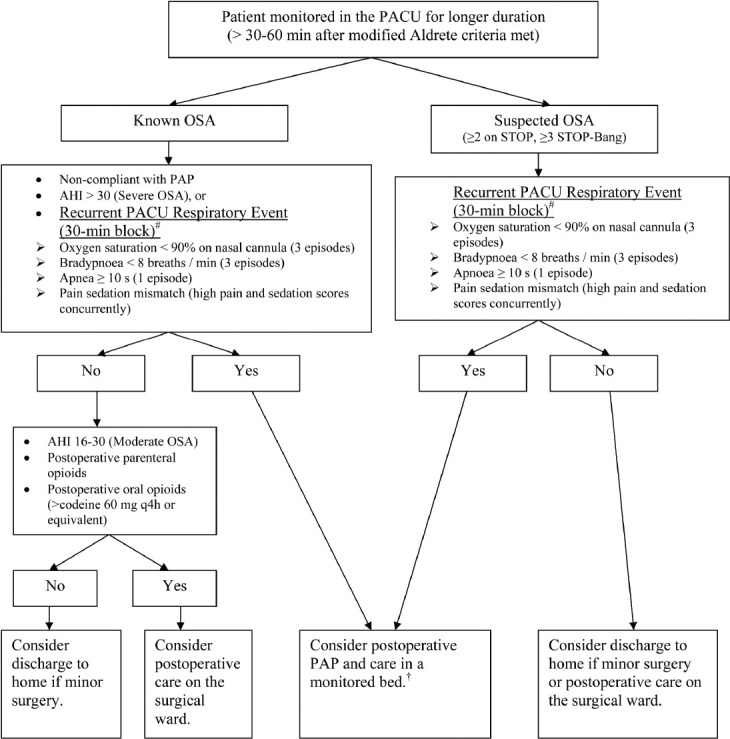
Postoperative management of the patient with confirmed or suspected obstructive sleep apnea (OSA) after general anesthesia. Adapted with permission from Seet and Chung [28] Figure 2 Legend: # Recurrent post-anesthesia care unit (PACU) respiratory events – any event that has occurred more than once in each 30-minute evaluation period (not necessarily the same event). †Positive airway pressure (PAP) therapy included continuous PAP, bilevel PAP, or auto-titrating PAP, and monitored bed was a setting with continuous oximetry and possible early nursing intervention (e.g., intensive care unit [ICU], step-down unit, or remote pulse oximetry with telemetry in the surgical ward). Pain-sedation mismatch = event with simultaneous high pain scores and oversedation.

In response to the gap in PACU discharge criteria, the Mayo Clinic introduced a system where surgical patients in the PACU are continuously assessed by nursing staff for signs of respiratory depression (episodes of respiratory depression are termed “*Respiratory Specific Events*”) [1]. *Respiratory specific events* are defined as hypoventilation (3 episodes of <8 respirations per minute); apnea (episode of apnea ≥10 seconds); hypoxemia (3 episodes of oxyhemoglobin desaturations as measured by pulse oximetry [<90% with or without nasal cannula]); and “pain/sedation mismatch” (defined as Richmond Agitation Sedation Score [24,25] of -3 to -5 with a numeric pain score >5 [from 0, no pain, to 10, worst pain imaginable]) [1]. Also, all patients are preoperatively screened for OSA by nursing query during the check-in process, and patients who deny OSA history are screened for such with validated assessment tools [26,27]. Patients who are observed to have a *respiratory specific event*, have their PACU extended for at least two 30-minute assessment periods to observe further signs of respiratory depression. Patients that show these signs or have either a history of untreated OSA or a positive screening are provided with additional postoperative monitoring on hospital wards. Based on the overall assessment of the patient, it could denote continuous pulse oximetry or admission to an advanced care ward with capabilities for more advanced monitoring (Figure 2) [1,28].

### Respiratory depression in PACU and subsequent complications – Mayo Clinic experience

**FIGURE 3:**
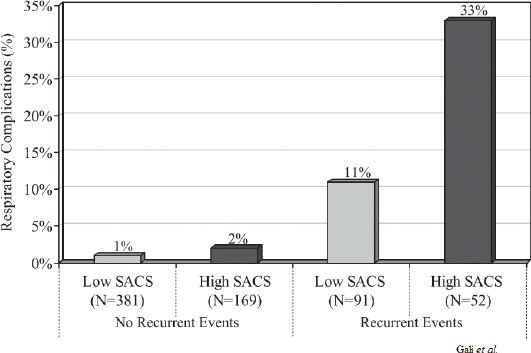
Postoperative respiratory complication rate and post-anesthesia care unit (PACU) respiratory recurrent event. With permission from Gali et al. [2].

*Respiratory specific events* are strongly associated with subsequent respiratory complications. Gali et al. [2] investigated patients undergoing inpatient surgical procedures with a length of stay greater than 48 hours, evaluating them based on their preoperative OSA rating and the recurrence of PACU events (e.g., apnea, bradypnea, oxyhemoglobin desaturations, and pain-sedation mismatch). Patients with both high OSA rating and recurrent events in the PACU had a respiratory complication rate of 33%, while patients with recurrent respiratory specific events but a low OSA rating had 3 times fewer complications. In contrast, patients that did not present recurrent events in the PACU had respiratory complications rate of ≤2%, regardless of OSA screen (Figure 3) [2].

Two separate analyses from the Mayo Clinic, from July 1, 2008 to June 30, 2010 (134 patients) [29] and July 1, 2011 to December 31, 2015 (128 patients) [4] of naloxone administration to reverse opioid-induced respiratory depression within 48 hours following PACU discharge to standard postoperative wards, both found a five-fold increased risk in patients who were witnessed to have a *respiratory specific event* in the PACU. Another study from the Mayo Clinic found that patients administered naloxone in the PACU were at increased risk for other postoperative complications (odds ratio 3.39 [95% confidence interval (CI) 2.22–5.23]) [30]. A secondary analysis of 199 patients who were witnessed to have respiratory depression in the PACU and were subsequently referred (for unrelated signs and symptoms) to a sleep center for evaluation of OSA found that 148 (74.4%) had abnormal sleep studies, therefore, were positive for either OSA or sleep disturbed breathing [31]. Furthermore, patients who have respiratory specific events in the PACU, have higher rates of unplanned intensive care unit admissions (odds ratio 5.32 [95% CI 4.22–6.71]) and take longer to achieve PACU discharge criteria (188 ± 54 minutes vs. 106 ± 45 minutes) [6]. Similarly, patients that presented respiratory depression in the PACU and required naloxone administration had similar delayed phase I anesthesia recovery (183 ± 88 minutes vs. 107 ± 59 minutes), and higher rates of postoperative morbidity (e.g., pneumonia [7.3% vs. 14.5%], myocardial infarction [2.2% vs. 0.4%]), and mortality (3.1% vs. 0.6%) [30].

### Risk factors for respiratory depression in PACU - Mayo Clinic findings

There have been several studies from the Mayo Clinic examining for risk factors for respiratory depression in the PACU [23,32]. These risk factors can be broadly categorized into intrinsic patient factors and surgical/anesthetic factors (Table 1). One study of 11,970 patients undergoing elective lower extremity total joint arthroplasty with peripheral nerve blocks under either spinal or general anesthesia, found documentation for respiratory depression in 2836 (23.7%) patients, with documentation of apnea in almost 80% of cases [6]. Respiratory depression was more common among patients who had general (31.2%) than spinal anesthesia (14.4%) [6]. OSA was found to be associated with increased risk following general anesthesia (odds ratio 1.26 [95% CI 1.08–1.48]), and lower body mass index for the spinal group. Extrinsic factors included the use of preoperative sustained-release opioids, preoperative gabapentin, higher intraoperative opioids, use of isoflurane, and knee surgery [6]. The second large study was of 8567 patients undergoing laparoscopic surgery, where 1311 patients (15.3%) were found to have respiratory depression with apnea consisting of almost 60% of these events [3]. Similar to the orthopedic study, this study found that low body mass index, longer surgical duration, administration of gabapentin, midazolam, and higher doses of intraoperative opioids were associated with increased risk of respiratory depression in the PACU [3]. To confirm the association between postoperative respiratory depression and preoperative gabapentin, a secondary analysis using propensity score matching was performed and indeed confirmed this association (odds ratio 1.26, [95% CI 1.02–1.58]) [3]. A subsequent study from that cohort performed a propensity-matched analysis comparing patients who received isoflurane versus other agents and found isoflurane use was associated with increased risk of respiratory depression [32]. The third study examined PACU outcome changes following a practice improvement initiative where routine use of midazolam and isoflurane was largely replaced to an anesthetic devoid of benzodiazepines and mostly desflurane anesthesia [23]. In that cohort, when compared to isoflurane, desflurane was associated with a decreased rate of respiratory depression in PACU (odds ratio 0.72, [95% CI 0.55–0.93]). In contrast, midazolam use trended towards association with PACU respiratory depression (odds ratio 1.27, [95% CI 1.00–1.60]) [23]. Another study from Mayo Clinic examined clinical factors associated with the use of naloxone in the PACU to reverse opioid-induced respiratory depression. Over 3 years, 413 patients were analyzed, with an incidence of naloxone reversal of 2.5 [95% CI 0.7–6.5] per 1000 anesthetics following general anesthesia. Factors associated with respiratory depression included OSA (odds ratio 1.74 [95% CI 1.22–2.48]), higher disease burden (American Society of Anesthesiologists (ASA) physical status ≥ III, odds ratio 1.44 [95% CI 1.08–1.92]), and larger doses of intraoperative opioids (odds ratio 1.22 [95% CI 1.12–1.33] per 10 mg intravenous morphine equivalents) [30].

**TABLE 1:**
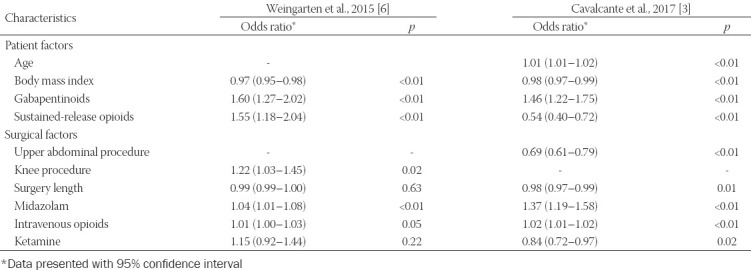
Patient and procedure characteristics associated with respiratory depression during anesthesia recovery

### Role of gabapentinoids in postanesthesia recovery – “friend or foe”?

The association between preoperative gabapentin and respiratory depression was an unexpected finding in several studies [17,18,33-37]. Toxicology literature suggests that ingestion of even large doses of gabapentin or pregabalin results in sedation, but not respiratory arrest [33-36]. Also, meta-analyses of preoperative gabapentin and pregabalin found an increased risk of postoperative sedation, but not respiratory depression [38,39]. However, randomized prospective trials and the resultant meta-analyses are known to underreport risk from real-world use [40-42]. In regards to the safety of gabapentinoids and its perioperative use, a prospective clinical trial evaluated the analgesic effect of perioperative pregabalin, remifentanil, and their combination. The trial found that pregabalin and remifentanil have an additive analgesic effect but also a substantial synergistic impact on respiratory drive [18]. A large population-based study from Toronto analyzed deaths among patients with non-malignant pain treated with chronic opioids and matched these cases using a 4:1 matching design with subjects similarly treated with chronic opioids for non-malignant pain, found associations with concomitant use of gabapentin and an increased risk of death. A follow-up study from that group found a similar relationship with pregabalin [17,37]. At the Mayo Clinic, it was found that home use of gabapentinoids had a dose-dependent association with naloxone administration in the wards, with an odds ratio of 2.64 [95% CI 1.31–5.35] for low-dose home use and 8.29 [95% CI 3.04–22.64] for high-dose home use. Maintenance of chronic gabapentinoids into the perioperative period was observed to be associated with an overall six-fold increased risk for naloxone administration, with a dose-dependent effect: odds ratio was 2.64 [95% CI 1.31, 5.35] and 8.29 [95% CI 3.04, 22.64] for low dose (300 mg) and high dose (600 mg) of gabapentinoids use, respectively, *p* ≤ 0.001 [4]. This effect was reflected in increased postoperative rapid response team activation, with an increased risk (odds ratio of 1.60 [95% CI 1.17–2.20]) of activation in patients who have preoperatively received gabapentin as a part of the enhanced recovery after surgery and anesthesia (ERAS) protocol [5]. Based on this emerging data, in December 2019, the FDA issued a warning on gabapentinoids usage and respiratory complications [7], increasing the recommended level of respiratory vigilance in patients with chronic use of this medication.

### Anesthetic management may improve recovery and reduce PACU complications

Reducing the incidence of postoperative respiratory depression in the PACU may decrease later episodes of severe postoperative adverse events. Using anesthetic techniques that enhance the speed of recovery may be beneficial. Some elements of anesthetic management directed at opioid-sparing suggested by enhanced recovery after major surgery [43] protocols could be useful. Pre-emptive treatment with acetaminophen, a low-cost and widely studied drug, can reduce the need for opiates, which in turn could reduce rates of opioid-induced respiratory depression [43].

Intraoperative management can further be optimized to facilitate early recovery considering propofol as the primary agent on induction [43], shorter-acting volatile anesthetics for maintenance, thoughtful (more restrictive) use of opioids [6,23,32], and consideration of regional or neuraxial anesthesia [15,43,44]. The use of benzodiazepines should be curtailed and reserved for those patients in need of anxiolysis [6]. The routine use of preoperative gabapentinoids needs to be reconsidered, especially in light of their limited opioid-sparing effect compared with the increased rate of respiratory depression [3,4,6]. Perioperative administration of caffeine in chronic users has shown enhancement in anesthesia emergence, with no severe side effects reported, however, this practice was still not widely adopted [45-47].

In the early phases of anesthesia recovery, all patients should be monitored for signs of decreased respiratory drive. Healthcare providers should be aware that early signs of respiratory depression are associated with increased risk for pulmonary complications on the wards.

## CONCLUSION

Postoperative respiratory depression can lead to catastrophic adverse events. The effects of residual anesthetics, sedating analgesics, sleep-related breathing disorders, and higher levels of comorbidity are risk factors for postoperative respiratory depression. Early signs of respiratory depression represent a substantial risk for later in-hospital adverse events. Multimodal forms of anesthetic and pain management can reduce the use of opioids and may contribute to reducing postoperative complications.

## References

[1] Gali B, Whalen FX, Gay PC, Olson EJ, Schroeder DR, Plevak DJ (2007). Management plan to reduce risks in perioperative care of patients with presumed obstructive sleep apnea syndrome. J Clin Sleep Med.

[2] Gali B, Whalen FX, Schroeder DR, Gay PC, Plevak DJ (2009). Identification of patients at risk for postoperative respiratory complications using a preoperative obstructive sleep apnea screening tool and postanesthesia care assessment. Anesthesiology.

[3] Cavalcante AN, Sprung J, Schroeder DR, Weingarten TN (2017). Multimodal analgesic therapy with gabapentin and its association with postoperative respiratory depression. Anesth Analg.

[4] Deljou A, Hedrick SJ, Portner ER, Schroeder DR, Hooten WM, Sprung J (2018). Pattern of perioperative gabapentinoid use and risk for postoperative naloxone administration. Br J Anaesth.

[5] Hardman MI, Kruthiventi SC, Schmugge MR, Cavalcante AN, Jensen JB, Schroeder DR (2020). Risk factors and outcomes of postoperative emergency response team activation:A matched case-control study. Crit Care Resusc.

[6] Weingarten TN, Jacob AK, Njathi CW, Wilson GA, Sprung J (2015). Multimodal analgesic protocol and postanesthesia respiratory depression during phase I recovery after total joint arthroplasty. Reg Anesth Pain Med.

[7] FDA, DSC (2019). FDA warns about Serious Breathing Problems with Seizure and Nerve Pain Medicines Gabapentin (Neurontin, Gralise, Horizant) and Pregabalin (Lyrica, Lyrica CR).

[8] Rose DK, Cohen MM, Wigglesworth DF, DeBoer DP (1994). Critical respiratory events in the postanesthesia care unit. Patient, surgical, and anesthetic factors. Anesthesiology.

[9] Kumar GV, Nair AP, Murthy HS, Jalaja KR, Ramachandra K, Parameshwara G (2012). Residual neuromuscular blockade affects postoperative pulmonary function. Anesthesiology.

[10] Barone CP, Pablo CS, Barone GW (2003). A history of the PACU. J Perianesth Nurs.

[11] American Society of Anesthesiologists (2004). Standards for Postanesthesia Care.

[12] Aldrete JA, Kroulik D (1970). A postanesthetic recovery score. Anesth Analg.

[13] Aldrete JA (1998). Modifications to the postanesthesia score for use in ambulatory surgery. J Perianesth Nurs.

[14] Fu ES, Downs JB, Schweiger JW, Miguel RV, Smith RA (2004). Supplemental oxygen impairs detection of hypoventilation by pulse oximetry. Chest.

[15] Lippe PM (2008). The decade of pain control and research. Pain Med.

[16] Frasco PE, Sprung J, Trentman TL (2005). The impact of the joint commission for accreditation of healthcare organizations pain initiative on perioperative opiate consumption and recovery room length of stay. Anesth Analg.

[17] Gomes T, Greaves S, van den Brink W, Antoniou T, Mamdani MM, Paterson JM (2018). Pregabalin and the risk for opioid-related death:A nested case-control study. Ann Intern Med.

[18] Myhre M, Diep LM, Stubhaug A (2016). Pregabalin has analgesic, ventilatory, and cognitive effects in combination with remifentanil. Anesthesiology.

[19] Singh M, Liao P, Kobah S, Wijeysundera DN, Shapiro C, Chung F (2013). Proportion of surgical patients with undiagnosed obstructive sleep apnoea. Br J Anaesth.

[20] Hamlin RJ, Sprung J, Hofer RE, Schroeder DR, Weingarten TN (2013). Obesity trends in the surgical population at a large academic center:A comparison between 1989-1991 to 2006-2008 epochs. Acta Chir Belg.

[21] Lee LA, Caplan RA, Stephens LS, Posner KL, Terman GW, Voepel-Lewis T (2015). Postoperative opioid-induced respiratory depression:A closed claims analysis. Anesthesiology.

[22] Silber JH, Williams SV, Krakauer H, Schwartz JS (1992). Hospital and patient characteristics associated with death after surgery. A study of adverse occurrence and failure to rescue. Med Care.

[23] Weingarten TN, Bergan TS, Narr BJ, Schroeder DR, Sprung J (2015). Effects of changes in intraoperative management on recovery from anesthesia:A review of practice improvement initiative. BMC Anesthesiol.

[24] Sessler CN, Gosnell MS, Grap MJ, Brophy GM, O'Neal PV, Keane KA (2002). The Richmond Agitation-Sedation scale:Validity and reliability in adult intensive care unit patients. Am J Respir Crit Care Med.

[25] Vasilevskis EE, Morandi A, Boehm L, Pandharipande PP, Girard TD, Jackson JC (2011). Delirium and sedation recognition using validated instruments:Reliability of bedside intensive care unit nursing assessments from 2007 to 2010. J Am Geriatr Soc.

[26] Flemons WW, Whitelaw WA, Brant R, Remmers JE (1994). Likelihood ratios for a sleep apnea clinical prediction rule. Am J Respir Crit Care Med.

[27] Chung F, Abdullah HR, Liao P (2016). STOP-bang questionnaire:A practical approach to screen for obstructive sleep apnea. Chest.

[28] Seet E, Chung F (2010). Management of sleep apnea in adults-functional algorithms for the perioperative period:Continuing professional development. Can J Anaesth.

[29] Weingarten TN, Herasevich V, McGlinch MC, Beatty NC, Christensen ED, Hannifan SK (2015). Predictors of delayed postoperative respiratory depression assessed from naloxone administration. Anesth Analg.

[30] Weingarten TN, Chong EY, Schroeder DR, Sprung J (2016). Predictors and outcomes following naloxone administration during Phase I anesthesia recovery. J Anesth.

[31] Elleby LM, Sprung J, Weingarten TN (2019). Postoperative respiratory depression may be related to undiagnosed sleep-disordered breathing or obstructive sleep apnea. Anesth Analg.

[32] Cavalcante AN, Gurrieri C, Sprung J, Schroeder DR, Weingarten TN (2018). Isoflurane and postoperative respiratory depression following laparoscopic surgery:A retrospective propensity-matched analysis. Bosn J Basic Med Sci.

[33] Andrews CO, Fischer JH (1994). Gabapentin:A new agent for the management of epilepsy. Ann Pharmacother.

[34] Klein-Schwartz W, Shepherd JG, Gorman S, Dahl B (2003). Characterization of gabapentin overdose using a poison center case series. J Toxicol Clin Toxicol.

[35] Schauer SG, Varney SM (2013). Gabapentin overdose in a military beneficiary. Mil Med.

[36] Wills B, Reynolds P, Chu E, Murphy C, Cumpston K, Stromberg P (2014). Clinical outcomes in newer anticonvulsant overdose:A poison center observational study. J Med Toxicol.

[37] Gomes T, Juurlink DN, Antoniou T, Mamdani MM, Paterson JM, van den Brink W (2017). Gabapentin, opioids, and the risk of opioid-related death:A population-based nested case-control study. PLoS Med.

[38] Doleman B, Heinink TP, Read DJ, Faleiro RJ, Lund JN, Williams JP (2015). A systematic review and meta-regression analysis of prophylactic gabapentin for postoperative pain. Anaesthesia.

[39] Mishriky BM, Waldron NH, Habib AS (2015). Impact of pregabalin on acute and persistent postoperative pain:A systematic review and meta-analysis. Br J Anaesth.

[40] Moore TJ, Furberg CD, Glenmullen J, Maltsberger JT, Singh S (2011). Suicidal behavior and depression in smoking cessation treatments. PLoS One.

[41] Moore TJ, Singh S, Furberg CD (2012). The FDA and new safety warnings. Arch Intern Med.

[42] Singh S, Loke YK (2012). Drug safety assessment in clinical trials:Methodological challenges and opportunities. Trials.

[43] Joshi GP, Kehlet H (2019). Postoperative pain management in the era of ERAS:An overview. Best Pract Res Clin Anaesthesiol.

[44] Machi A, Joshi GP (2019). Interfascial plane blocks. Best Pract Res Clin Anaesthesiol.

[45] Fong R, Khokhar S, Chowdhury AN, Xie KG, Wong JH, Fox AP (2017). Caffeine accelerates recovery from general anesthesia via multiple pathways. J Neurophysiol.

[46] Fong R, Wang L, Zacny JP, Khokhar S, Apfelbaum JL, Fox AP (2018). Caffeine accelerates emergence from isoflurane anesthesia in humans:A randomized, double-blind, crossover study. Anesthesiology.

[47] Warner NS, Warner MA, Schroeder DR, Sprung J, Weingarten TN (2018). Effects of caffeine administration on sedation and respiratory parameters in patients recovering from anesthesia. Bosn J Basic Med Sci.

